# Application of high resolution melting assay (HRM) to study temperature-dependent intraspecific competition in a pathogenic bacterium

**DOI:** 10.1038/s41598-017-01074-y

**Published:** 2017-04-20

**Authors:** Roghaieh Ashrafi, Matthieu Bruneaux, Lotta-Riina Sundberg, Katja Pulkkinen, Tarmo Ketola

**Affiliations:** 1 Department of Biological and Environmental Science (and Nanoscience Center), University of Jyvaskyla, Centre of Excellence in Biological Interactions, P.O. Box 35, FI-40014 Jyvaskyla, Finland; 2 Department of Biological and Environmental Science, University of Jyvaskyla, P.O. Box 35, FI-40014 Jyvaskyla, Finland

## Abstract

Studies on species’ responses to climate change have focused largely on the direct effect of abiotic factors and in particular temperature, neglecting the effects of biotic interactions in determining the outcome of climate change projections. Many microbes rely on strong interference competition; hence the fitness of many pathogenic bacteria could be a function of both their growth properties and intraspecific competition. However, due to technical challenges in distinguishing and tracking individual strains, experimental evidence on intraspecific competition has been limited so far. Here, we developed a robust application of the high-resolution melting (HRM) assay to study head-to-head competition between mixed genotype co-cultures of a waterborne bacterial pathogen of fish, *Flavobacterium columnare*, at two different temperatures. We found that competition outcome in liquid cultures seemed to be well predicted by growth yield of isolated strains, but was mostly inconsistent with interference competition results measured in inhibition tests on solid agar, especially as no growth inhibition between strain pairs was detected at the higher temperature. These results suggest that, for a given temperature, the factors driving competition outcome differ between liquid and solid environments.

## Introduction

Climate models predict that climate change will lead to increased temperatures and larger thermal fluctuations in the future^1^. As a consequence of climate-induced range shifts and expanded human activities leading to the transportation of species, we expect to see previously separated populations come into contact with one another, providing new opportunities for interactions. Hence, it is of the utmost important to note that the effects of climate change can invoke factors other than temperature, particularly changes in biotic interactions within and among species^2–8^. For example, winners judged by the suitability of habitat can be competitively excluded by superior competitors^9, 10^. Given the global significance of pathogens, predicting how climate warming changes the outcome of intraspecific interactions in pathogens, which in turn would affect their population dynamics such as growth and virulence, is extremely important for the prevention of pathogen spread and the diseases caused by them.

Empirical investigations of intraspecific interactions using multiple strains from a single species have limitations in tracking individual strains in mixed cultures. Traditionally, genotyping or phenotypic markers based on LacZ, fluorescence or antibiotic resistance have been used discriminate strains and determine the relative abundance of multiple strains that interact within a mixed culture^11, 12^. However, markers based on genetic engineering may provide misleading results, as they may incur fitness costs or benefits in bacteria^13–15^. To address these problems, and in order to perform our study, we applied a method for the quantification of competition outcome for our study species, a bacterial fish pathogen, *Flavobacterium columnare*. We implemented an assay based on high-resolution melting curve (HRM) which has been used for various applications, most commonly the determination of DNA methylation status and genotyping^16–18^. Using genomic DNA containing a naturally occurring mutation in the tryptophan synthase gene *trpB* enabled us to quantitatively distinguish several *F*. *columnare* strains belonging to two genotypes of *trpB* from mixed-genotype competition cultures. To our knowledge, this is the first study that has used a HRM approach to monitor and quantify very closely related genotypes of one bacterial species in a single tube, and the method is extendable to multiple genotypes by multiplexing, as is exemplified in Appendix S1 (See also Fig. S4).

*F*. *columnare* serves as an excellent model system to examine whether temperature-dependent biotic interactions, such as competition, could alter population dynamics and climate warming projections for a few reasons. First, it has been shown that high summer temperatures correlate with increased *F*. *columnare* outbreaks^19^. Second, multiple genotypes are commonly present in a single region^20, 21^ implying the potential for intraspecific competition among genotypes. Third, competition among *F*. *columnare* strains, especially between the ones chosen for this experiment, is not restricted to resource competition and strains can directly interfere with competitors via growth inhibition^21, 22^.

The aim of our study was to test if head to head competition, interference competition, and growth parameters in two temperatures will lead to the same or different conclusions. If for the majority of the clone pairs the competition outcomes in different temperatures can be predicted by growth assays or interference assays that will facilitate predictions of future climate change on this important fish pathogen. However, if predictions between growth assays, interference, and head to head competition drastically differ, it will cause challenges in predicting which of the *F*. *columnare* genotypes will excel under progressing climate change. Although we use here a species with applied importance, the same question applies to all attempts to understand the fate of species under changing conditions in the wild, where intraspecific competitive interactions are the norm rather than the exception.

## Results

### Quantification of genotype proportions in control mixtures using HRM

**Figure 1:**
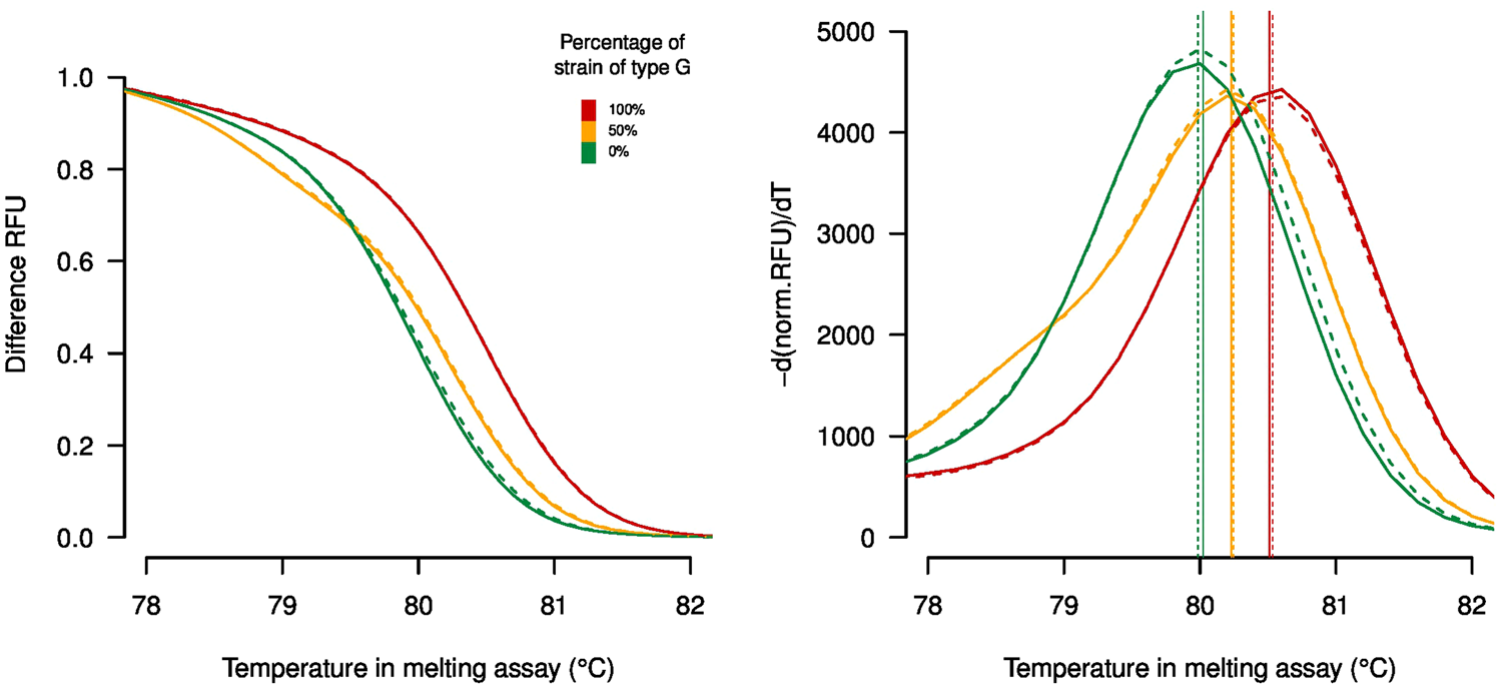
High-resolution DNA melting curve analysis results for a 97-bp amplicon containing one SNP in *trpB* gene for *Flavobacterium columnare*. (Right) Normalized melting curves, (Left) derivative plots. The melting curves depict pure genotype A, pure genotype G, and 50/50 ratio of both genotypes. Solid and dashed lines represent replicate measurements.

A single SNP (T > G substitution) at position 222 of the *trpB* gene was used to implement the high-resolution melting analysis (HRM) for *F*. *columnare* genotypes, G and A, circulating in Finnish fish farms. The amplicon subjected to high resolution melting analysis could be used to distinguish between the two SNP alleles based on the melting curve shape. The melt peak of the genotype A was centered at 80 °C, while that of the genotype G was centered at 80.60 °C (Fig. 1). Sequencing of the amplified products in both directions confirmed the HRM results (Fig. S2).

**Figure 2:**
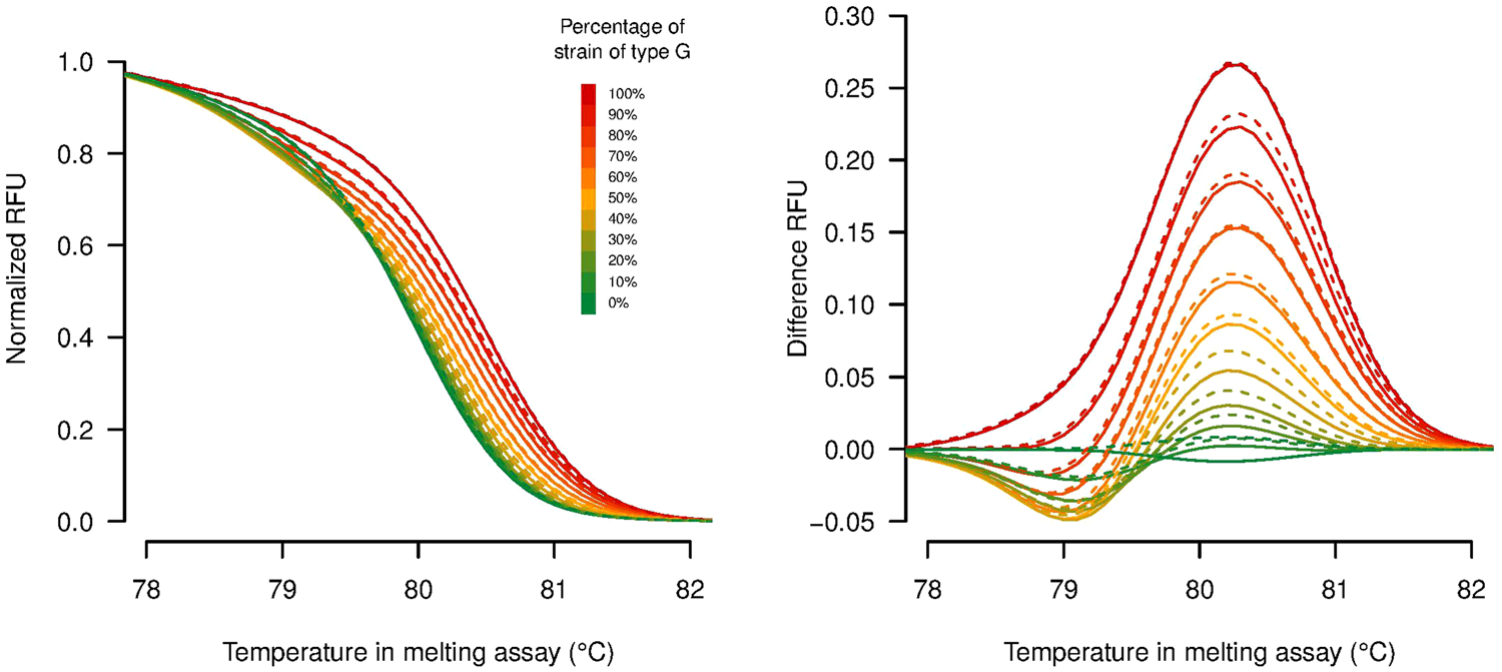
(Right) Normalized melting curves and (Left) difference plots of reference samples consisting of purified DNA from genotypes G and A pooled in known proportions (13 different proportions of the two genotypes: 100%, 95%, 90%, 80%, 70%, 60%, 50%, 40%, 30%, 20%, 10%, 5% and 0%). Solid and dashed lines represent replicate measurements.

Comparison of the melting temperature values for the reference samples across the HRM runs showed that there were some variations between the runs. For this reason, it is important to use within-plate reference samples for the calibration of each run. The within-plate calibration curves showed a good linear fit between melting temperatures and genotype proportions for the calibration samples (see R^2^ values on Fig. S3). For competition group C, there was a technical issue during the preparation of the reference samples for mixes from 20% to 60%, and those calibration samples were discarded from the analysis. Our estimate of genotype proportions were consistent with the clustering results obtained using the Precision Melt Analysis software (Bio-rad); however, they enabled us to obtain a more quantitative estimate of genotype proportions in the experimental samples compared to the cluster classification obtained with Precision Melt Analysis software (Fig. 2).

### Competition and growth

**Figure 3:**
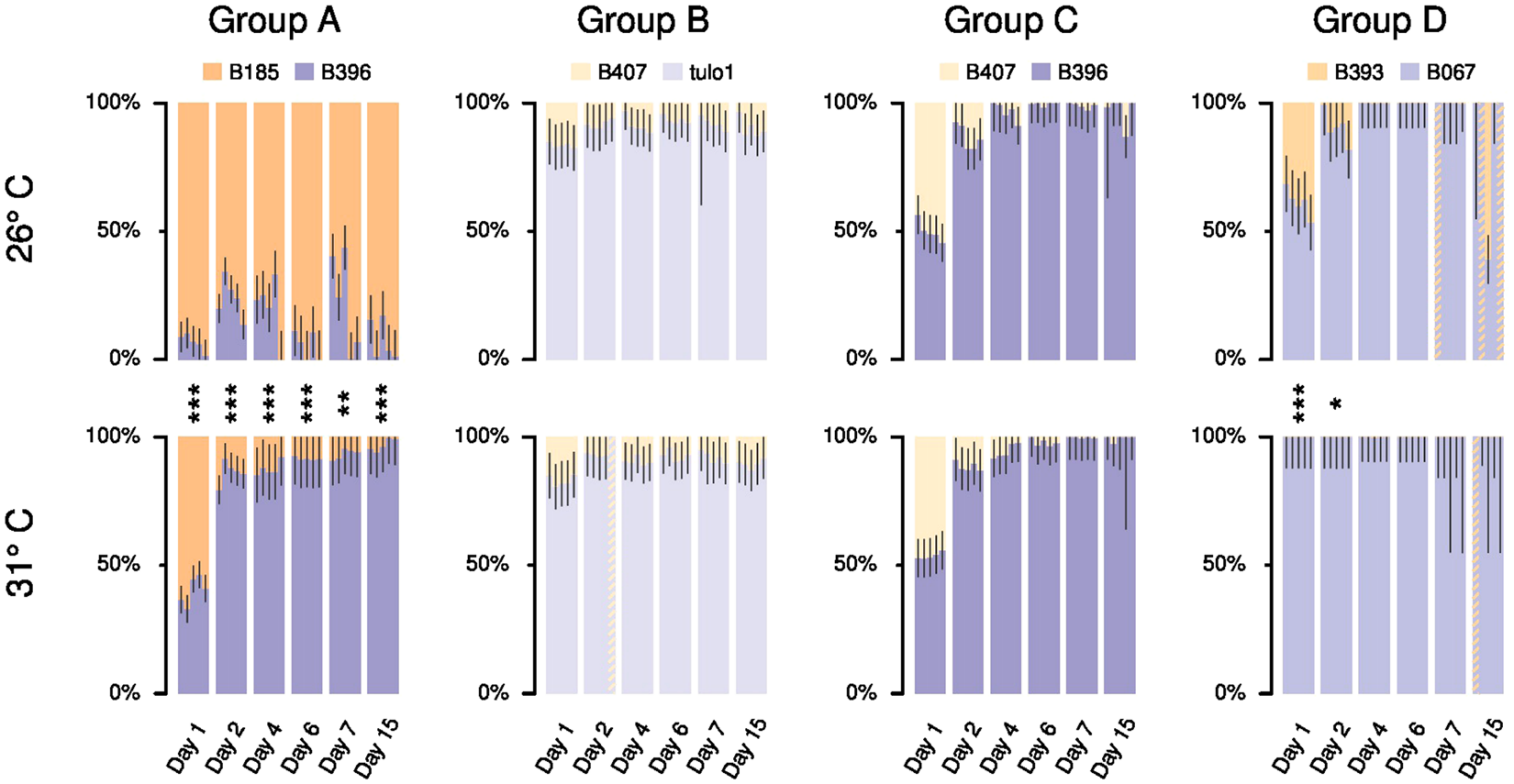
Genotype proportions estimated by HRM runs after 1, 2, 4, 6, 7 and 15 days of competition in liquid medium. For each day, vertical bars show the relative proportions of each competing genotype in five experimental replicates within each competition group (A, B, C and D) and black lines indicates the 90% confidence interval for each proportion estimate based on the triplicate HRM samples. The ordering of experimental replicates in the bar plots are the same from day 1 to day 15 within each group × temperature treatment. The names of the competing genotypes are indicated on the vertical axes. Hatched bars indicate replicates for which HRM data could not be used to estimate genotype proportions. For each day within each competition group, symbols between the 26 °C and 31 °C bar plots indicate the p-values of a Welch’s t-test comparing the genotype proportions for the 26 °C replicates and for the 31 °C replicates (*** < 0.001 < ** < 0.01 < * < 0.05).

The temporal change of genotype proportions as estimated by HRM during the competition in liquid medium is presented in Fig. 3. Overall, the five replicates within each competition group showed consistent results. Group A was the only competition group for which the outcome of competition differed between 26 °C and 31 °C (Table 1). The strains with the faster growth and higher yield were those able to utilize resources faster and won in all tested competition pairs at both temperatures, except in one group (C) where the losing competitor had a higher yield than the winner at 26 °C. However, the results of interference competition on plates with solid agar matched the results of the competition in liquid media in only half of the cases (Table 1). At 31 °C no inhibition between strains was detected (Table 1).

**Table 1:** Details of *Flavobacterium columnare* strains used in this study, and their performance in competition pairs (A–D, + indicates winner strain in competition).

Strains	Genotype	Year	Origin	Competition Group	26 °C	Growth	Yield	Inhibition	31 °C	Growth	Yield	Inhibition
					Comp.				Comp.			
B185	G	2009	Fish farm	A	+	**0**.**199** ± **0**.**020** (**5**)	**0**.**788** ± **0**.**088** (**5**)	−	−	0.199 ± 0.012 (4)	0.198 ± 0.006 (4)	No inhibition
B396	A	2010	Fish farm		−	0.158 ± 0.028 (5)	0.699 ± 0.052 (5)	+	+	**0**.**200** ± **0**.**010** (**4**)	**0**.**298** ± **0**.**038** (**4**)	No inhibition
B407	G	2010	Wild	B	−	0.138 ± 0.038 (5)	0.717 ± 0.054 (5)	−	−	0.184 ± 0.044 (5)	0.137 ± 0.022 (5)	No inhibition
TULO1	A	2010	Fish farm		+	**0**.**220 ± 0**.**022** (**3**)	**0**.**779 ± 0**.**106** (**3**)	+	+	**0**.**208 ± 0**.**022** (**5**)	**0**.**285 ± 0**.**068** (**5**)	No inhibition
B407	G	2010	Wild	C	−	0.138 ± 0.038 (5)	**0**.**717 ± 0**.**054** (**5**)	−	−	0.184 ± 0.044 (5)	0.137 ± 0.022 (5)	No inhibition
B396	A	2010	Fish farm		+	**0**.**158 ± 0**.**028** (**5**)	0.699 ± 0.052 (5)	+	+	**0**.**200 ± 0**.**010** (**4**)	**0**.**298 ± 0**.**038** (**4**)	No inhibition
B393	G	2010	Wild	D	−	0.189 ± 0.030 (5)	0.622 ± 0.046 (5)	+	−	0.190 ± 0.022 (5)	0.166 ± 0.006 (5)	No inhibition
B067	A	2007	Fish farm		+	0.189 ± 0.010 (5)	**0**.**773 ± 0**.**064** (**5**)	−	+	**0**.**216 ± 0**.**016** (**5**)	**0**.**347 ± 0**.**070** (**5**)	No inhibition

Growth and yield equals growth rate and yield measured in liquid medium, and inhibition (+) refers to which strain inhibited which in competition pairs.

In bold: indicates which strains of a given pair has the fastest growth rate or highest yield at a given temperature.

## Discussion

While temperature change can affect thermal sensitivities, tolerance range, and optimal performance of populations, predicting how this change will ultimately affect the structure and distribution of species depends also on indirect effects mediated by biotic interactions^5^. However, due to technical limitations, such as tracking individual isolates in co-cultures, relatively few studies have empirically examined how joint changes in intraspecific interactions and temperature affect ecological responses to increasing temperatures^12^. Based on a diagnostic SNP, we successfully established a method based on real-time PCR and HRM analysis for the rapid differentiation and quantification of two closely related genotypes of *F*. *columnare* in mixed culture. Using this method, we could resolve the outcome of head-to-head competition of bacterial strains in liquid culture. Since this method does not require any alteration in the genotype of the wild-type strain, it is a suitable method to study ecological fitness and competitive interactions between natural isolates. Overall, our results confirm that the HRM method can be used to accurately quantify the relative abundance of different strains in co-culture samples, and suggest that HRM offers a simple and cost effective means for detection of these strains in monitoring intraspecific competition in a single culture tube.

We observed that the competition outcomes of two strains in mixed culture at two temperatures were associated with changes in bacterial growth rate in the given temperature, rather than with their capacity for interference (Table 1). This gives a positive indication that growth measurements could be effective in predicting the success of the clones in liquid cultures and in different temperatures, despite the interference competition between genotypes of this species. This verification of the close match between growth parameters and competition is important to model possible outbreak scenarios under changing environmental conditions in environments where multiple pathogen strains co-occur.

Bacteria often produce toxins that kill or inhibit the growth other strains^23–25^. Inconsistency between competition outcomes in liquid cultures and on plate culture observed in our study suggests that benefits of interference on a surface is relaxed in liquid culture, where the bacterial cells are more likely to have less frequent contact with competitor cells, and the toxins are subjected to dilution. This indicates different trajectories of competition strategies depending whether the cells live in biofilm or in a planktonic state^26, 27^. Toxins are, indeed, commonly released in surfaces (such as biofilm) in which bacterial populations are dense and consequently nutrient limited^28^. Furthermore, limited dispersal can favour toxin production, suggesting that this mechanism will profit bacteria growing on surfaces rather than the planktonic cells in free waters and liquid cultures^29^.

Increased global temperatures can strongly decrease the efficiency of food production by influencing pathogen species^30^, such as *F*. *columnare* ^19, 21, 31^. Prolonged infective seasons provided by the higher mean temperatures allow for more bacterium-bacterium and bacterium-host interactions, increasing the benefits of interference. Increased frequency of high and maximum temperatures, on the other hand, may select for bacterial strains with a wider temperature optimum, and change the strain composition of bacterial populations. Interestingly, however, we did not find any interference at high temperatures (31 °C). Technical problems can be safely ruled out as interference tests at both temperatures were conducted at the same time, from the same original bacterial stocks. Previous studies have shown that bacteriocin (growth-inhibiting substance needed for interference) production is sensitive to high temperature in lactic acid bacteria and in *Yersinia* ^32–34^. In temperate regions, the temperature of the aquatic environment rarely exceeds the +25 °C used as the lower temperature in our interference experiments, which could maintain diversity and competitive interactions in bacterial populations. For example, during recent years the propensity of *F*. *columnare* strains isolated from disease epidemics from fish farms to inhibit the growth of competing strains has increased, suggesting increased intraspecific competition in the fish farming environment^21^. The benefits of interference in *F*. *columnare* might be realized in the field conditions during biofilm formation and multiple infections, which are likely to occur when several strains are present.

Most of the current research on the effects of climate change concentrates on single genotypes, or species in isolation, but in the wild the situation is not so simple. Although laboratory experiments, such as ours, can shed light on the pairwise interactions of bacterial strains and improve our understanding on traits that make certain strains more successful, conditions in the environment are more complex. In real life, species and genotypes interact and may have additive effects for factors such as virulence^35^, thus proxies of fitness, such as individual growth rates, might not be predictive enough of which of the genotypes will excel in competition. The use of the HRM method with high sensitivity and specificity will likely allow us mimic more natural systems, which will in turn help in understanding the circumstances that affect pathogenicity of this environmentally growing opportunistic bacterium. Moreover, as shown in Appendix S1 (See also Fig. S4), this method is extendable to simultaneous quantitative separation of multiple genotypes. We expect this method to be useful in disentangling how global warming and other abiotic and biotic factors contribute to bacterial interactions and impact the dynamics and functionality of bacterial populations.

## Material and Methods

### Bacterial strain characteristics and culture conditions

Six *F*. *columnare* isolates, previously assigned to two genetic groups (genotypes G or A) with the MLSA method^20^, were used in this study. Before growth yield measurement, inhibition and competition, the strains were revived from the frozen stocks to 3 ml of Shieh medium^36^ and incubated for 24 h in a shaker (200 rpm) at 26 °C. After 24 hours, cultures were diluted into fresh modified Shieh medium (1:10) and allowed to regrow at 26 °C on the shaker for another 24 hours. The six *F*. *columnare* isolates were distributed into four competition pairs, each containing one strain from the G- and one strain from the A- genotype, and all different growth and competition assays were performed on these strains (Table 1).

### Competition

The optical density (OD, at 570 nm) of the bacterial cultures was measured with a spectrophotometer adjusted to an approximate value of 0.140 (~7.6 × 10^6^ cells/ml) using the Shieh medium. Then, replicates made from 250 µl of pre-culture from a genotype A strain and 250 µl of pre-culture from a genotype G strain mixed in 9.5 ml of Shieh medium were put in two different temperatures (26 °C and 31 °C, 5 replicates per temperature). After 24 hours, the overnight competition mixture was diluted 100-fold (0.1 ml into 9.9 ml) into fresh medium, and this was repeated every 24 hours for 14 days. Total DNA was extracted immediately after mixing the pre-cultures at the start of the experiment (day 0) and from overnight cultures prior to dilution into fresh medium on day 1, 2, 4, 6, 7, and 15. The extracted DNA was stored at −20 °C until further analysis with HRM assay (below).

### HRM assay development

HRM analysis uses a saturating, double-stranded DNA-binding dye, which specifically intercalates with double stranded DNA. In the post-PCR step, when the dsDNA dissociates into single strands, there is no longer any double stranded DNA present and thus fluorescence is reduced. The change in fluorescence is plotted against the temperature, generating a melting curve characteristic of the amplicon^16, 17^. The six strains used in this study (Table 1) served as controls for developing the HRM assay based on real-time PCR associated with melting curve analysis in order to quantify the proportions of strains of G genotype and strains of A genotype in a mixed culture. These two genotypes exhibited three single-nucleotide polymorphisms (SNPs) in the *trpB* gene, in positions 222, 587, and 590 of the amplified DNA fragments of the gene (NCBI Reference Sequence: LN624115, LN624122)^20^. HRM assay was not able to correctly discriminate between targeted genotypes and amplicons containing two or more SNPs. In this study, therefore, we describe the development of a real-time PCR-HRM technique targeting only one SNP (position 222) for rapid discrimination between the two genotypes, as described below. Extension of this method to discriminate between several genotypes, by multiplexing, is described in Appendix S1 (See also Fig. S4).

### DNA extraction

Bacterial cells were harvested from overnight cultures from the competition experiment by centrifugation at 6000 g for 10 min at room temperature, and genomic DNA was extracted using the Wizard Genomic DNA Purification Kit (Promega, USA). The DNA concentration and purity after extraction was estimated using the Qubit® Fluorometer (Life Technologies, Carlsbad, USA). DNA quality was assessed on an agarose gel and the final concentration was adjusted to 5 ng µl^−1^.

### Primer design and PCR amplification

Forward (5′-CGCAGAAGTCCGTCCTG-3′) and reverse (5′-AAAGGTATCTAGCGGATTGTT-3′) primers were designed to target 97 bp of the *trpB* gene (bp 171–267), spanning a single SNP (T > G substitution) at position 222 of the gene. This primer was designed using the Primer-BLAST software (http://www.ncbi.nlm.nih.gov/tools/primer-blast/) (Fig. S1). Optimal primer annealing temperatures were established by temperature gradient PCR and HRM.

PCR amplification and HRM analysis were performed using CFX Real Time PCR Detection System (Bio-Rad Laboratories, USA). The PCR amplifications were performed and monitored using the CFX Manager Software, and the HRM data was analyzed with the Bio-Rad Precision Melt Analysis. PCR amplification was performed in a total volume of 10 *μ*L, containing 5 μL of 1x Precision Melt Supermix (Bio-Rad Laboratories, USA), 1 *μ*L 200 nM of each designed primer, and 4 *μ*L 5 ng µl-1 of the DNA template. All samples were amplified in triplicate. The PCR reaction started at 95 °C for 1 minute for initial denaturation, followed by 40 cycles of 30 seconds at 95 °C for denaturation, 30 seconds at 63 °C for annealing and another 30 seconds at 72 °C for extension. The PCR amplification was then followed by heteroduplex formation by heating at 95 °C for 30 seconds and subsequent cooling at 60 °C for 1 minute. The high-resolution melting analysis was performed immediately afterwards by increasing the temperature from 65 °C to 95 °C by steps of 0.2 °C maintained for 10 seconds each.

### Primer efficiency

The primer efficiency was assessed using both G and A genotypes using 10-fold serial dilutions of DNA of each genotype. The slopes of the standard curves (an indicator of PCR efficiency) were similar, ranging from −3.45 (genotype A) to −3.56 (genotype G), demonstrating that the assays should be able to detect each genotype with similar high efficiencies ranging from 94.8% (genotype A) to 90.8% (genotype G).

### Sequencing of targets

Real-time PCR products were subsequently sequenced to verify the identity of the amplified sequences. Sanger sequencing was performed on the same amplicons as used for HRM analysis. 5 μl of PCR products were purified with exonuclease I and Fast-AP (Thermo Fisher Scientific, Waltham, USA) for 15 min at 37 °C and 15 min by 80 °C. A sequencing reaction was set up with 1 μl of purified PCR products and the BigDye® Terminator v1.1 Cycle Sequencing Kit (Life Technologies). Briefly, each 20 μl sequencing reaction mixture contained 1 μl of PCR amplicon, 0.16 μM of either forward or reverse PCR primer, 0.5 μl of BigDye Ready Reaction Mix, and 1 X sequencing buffer. The sequencing reaction conditions were as follows: 30 cycles of denaturing at 96 °C for 10 seconds, annealing at 50 °C for 5 seconds, and extension at 60 °C for 4 minutes. The sequencing products were purified using ethanol/EDTA/sodium acetate precipitation. Sequencing was performed on an ABI 3130xl 16-capillary automated genetic analyser.

### Quantification of mixed genotypes in competition cultures

For each HRM run, reference samples consisting of purified DNA from genotypes G and A pooled in known proportions (13 different proportions of the two genotypes: 100%, 95%, 90%, 80%, 70%, 60%, 50%, 40%, 30%, 20%, 10%, 5% and 0%) were included in duplicate in the plate, and a no template control (NTC) was included in triplicate in the plate. Samples from the competition experiment for which genotype proportions were to be determined were run in triplicate. For each well, melting temperature was defined as the temperature of the inflexion point of the melting curve, using normalized relative fluorescent units (RFU). This point was determined by searching for the minimum value of the first-order derivative of the melting curve. Within each plate, the reference samples were used to calibrate the relationship between genotype proportion and melting temperature and to estimate the genotype proportions in the experimental samples. A linear model of the form [*meltingTemp* = *a* + b * genotypeProp] was fitted to the calibration data. The genotype proportions in the experimental samples were estimated by using a piecewise-defined function using the relation [*genotypeProp* = *1*/b * (*meltingTemp* − *a*)] and setting the estimated values to 0% if they were negative and 100% if they were greater than 100% (Fig. S3). Prior to genotype proportion estimation experimental sample, wells for which the melting temperature was outside the range of values observed for calibration samples by more than 10% the calibration range span, were discarded. Confidence intervals were calculated according to Lavagnini *et al*.^37^.

### Growth

The growth rate and yield of isolates were measured in liquid culture using a temperature-controlled spectrophotometer (Bioscreen C, Growth Curves Ltd, Helsinki, Finland)^21^. Briefly, after isolate revival at 26 °C for 24 h on a shaker (120 rpm.), the optical density (OD, at 570 nm) of the bacterial cultures was measured with a spectrophotometer adjusted to same level (0.10–0.20). After OD adjustment, 40 µl of each isolate was inoculated into 400 µL of fresh Shieh liquid medium on a Bioscreen C 100 wells plates in five replicates and randomized order. Plates were placed in Bioscreen at 26 °C and 31 °C for 4 days until growth in all wells stopped. To find the maximum growth rate (OD460–580 nm h^−1^) and maximum population size (yield), we estimated the biomass with a MATLAB (version 2008b; MathWorks Inc., Natick, MA) script that fits linear regressions into ln-transformed population growth data consisting of 30 datapoints’ sliding time window. The MATLAB code to perform these analyses is further described Ketola *et al*.^38^.

### Inhibition assays

Within each of the above mentioned competition groups, the inhibitory activity of *F*. *columnare* strains was tested reciprocally using an inhibition zone method with four replicates per assay, and a double layer method^21^. The optical density (OD, at 570 nm) of the bacterial cultures was first adjusted to same level (0.250–0.290). Fresh overnight-grown ‘recipient’ bacterial cultures in about 300 µl were mixed with 3 ml of molten soft Shieh agar (47 °C), which was then poured on to the surface of dried Shieh agar. Five microliters of the unfiltered supernatant of the ‘donor’ cultures, which has been centrifuged at 17 000 g for 3 min in room temperature, were spotted on the surface of the top agar. Plates were incubated for 48 h either at 26 °C or 31 °C; then, the plates were checked to determine whether the ‘donor’ strain had caused a growth inhibition of the underlying ‘recipient’ bacterial lawn.

### Data Accessibility

DNA sequences used to develop the primers: NCBI Reference Sequence: LN624115, LN624122. Melting temperatures and calibration curve for HRM runs in supplementary material: Fig. S3. Data from HRM measurements (melting curves from controlled mixes of genotypes and melting temperatures for experimental and calibration samples from the competition experiment) were deposited in JYx and are publicly available at https://jyx.jyu.fi/dspace/handle/123456789/53541.

## Electronic supplementary material

supplementary material
