# Supplementary material for: Application of high resolution melting assay (HRM) to study temperature-dependent intraspecific competition in a pathogenic bacterium
Source: Sci Rep. 2017 Apr 20;7:980. doi: 10.1038/s41598-017-01074-y (PMC5430548; doi:10.1038/s41598-017-01074-y)
Supplement: Supplementary file 1 — supplementary material [file 41598_2017_1074_MOESM1_ESM.pdf]

SUPPLEMENTARY MATERIAL

Application of high resolution melting assay (HRM) to study temperature-dependent intraspecific competition in a pathogenic bacterium.

Roghaieh Ashrafi, Matthieu Bruneau, Lotta-Riina Sundberg, Katja Pulkkinen, Tarmo Ketola

Figure S1. Alignment of Forward and reverse primers that were designed to target 97 bp of the trpB gene (bp 171–267), spanning a single SNP (T>G substitution) at position 222 of *trpB* gene.

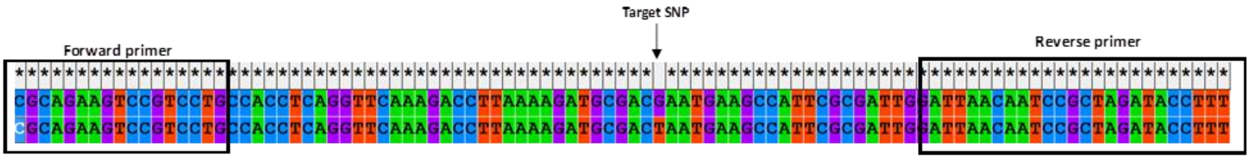

Figure S2. Chromatogram of reference samples by Sanger sequencing. 13 different proportions of the two genotypes: 100%, 95%, 90%, 80%, 70%, 60%, 50%, 40%, 30%, 20%, 10%, 5% and 0%.

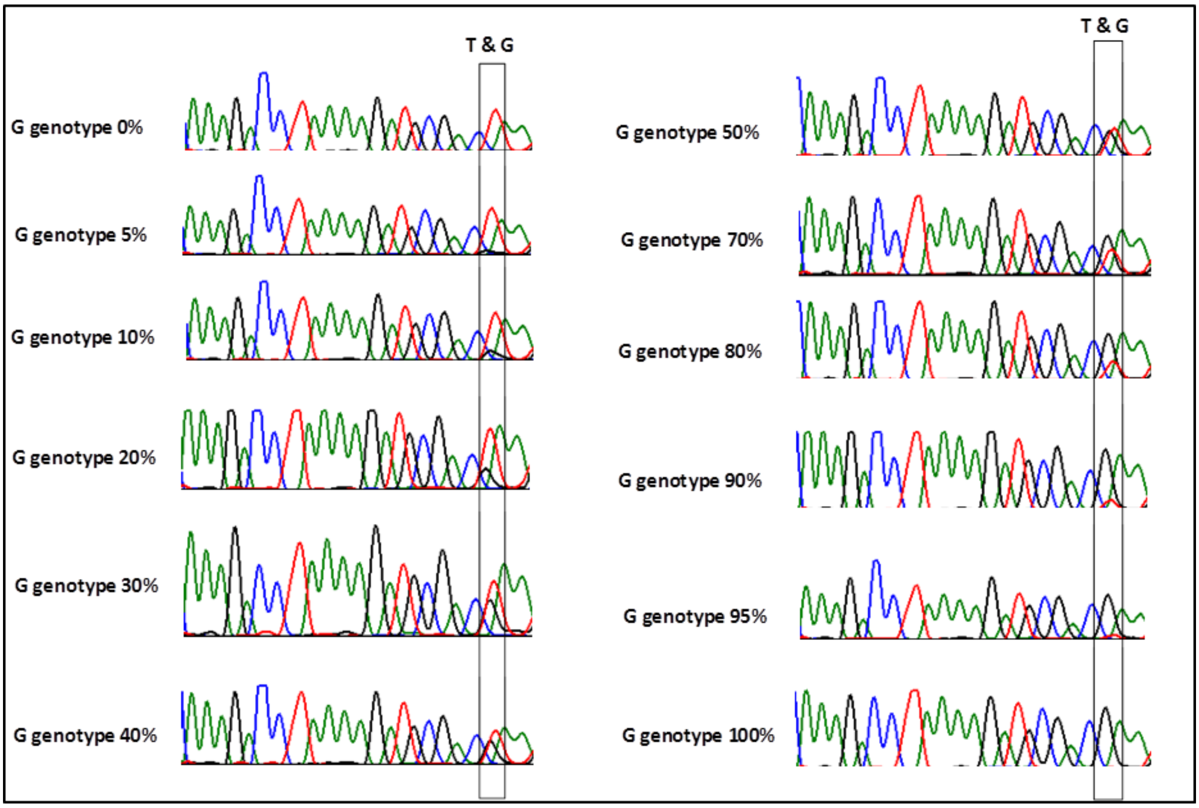

Figure S3. Melting temperatures and calibration curve for HRM runs. Each horizontal pair of panels corresponds to one run. Plate run name is indicated as the title of left panels (“A01”, “A35,” ...). Left panels, melting temperature values observed for each plate well. Experimental samples are marked with blue and calibration sample of known genotype proportions marked with red. Dashed lines indicate the range of melting temperature values observed for calibration samples +/- 10% of the range span. Wells for which melting temperature values were outside this range (blue crosses) were discarded from the analysis. Right panels, calibration curve used to estimate genotype proportions in experimental samples. Red dots are calibration samples. R^2 values of the linear model *proportion ~ meltingTemp* fitted on the calibration sample data are reported. Estimated genotype proportions for experimental samples which were below 0 or above 1 according to the linear fit model were set to 0 or 1, respectively, as shown by the plotted piecewise function.

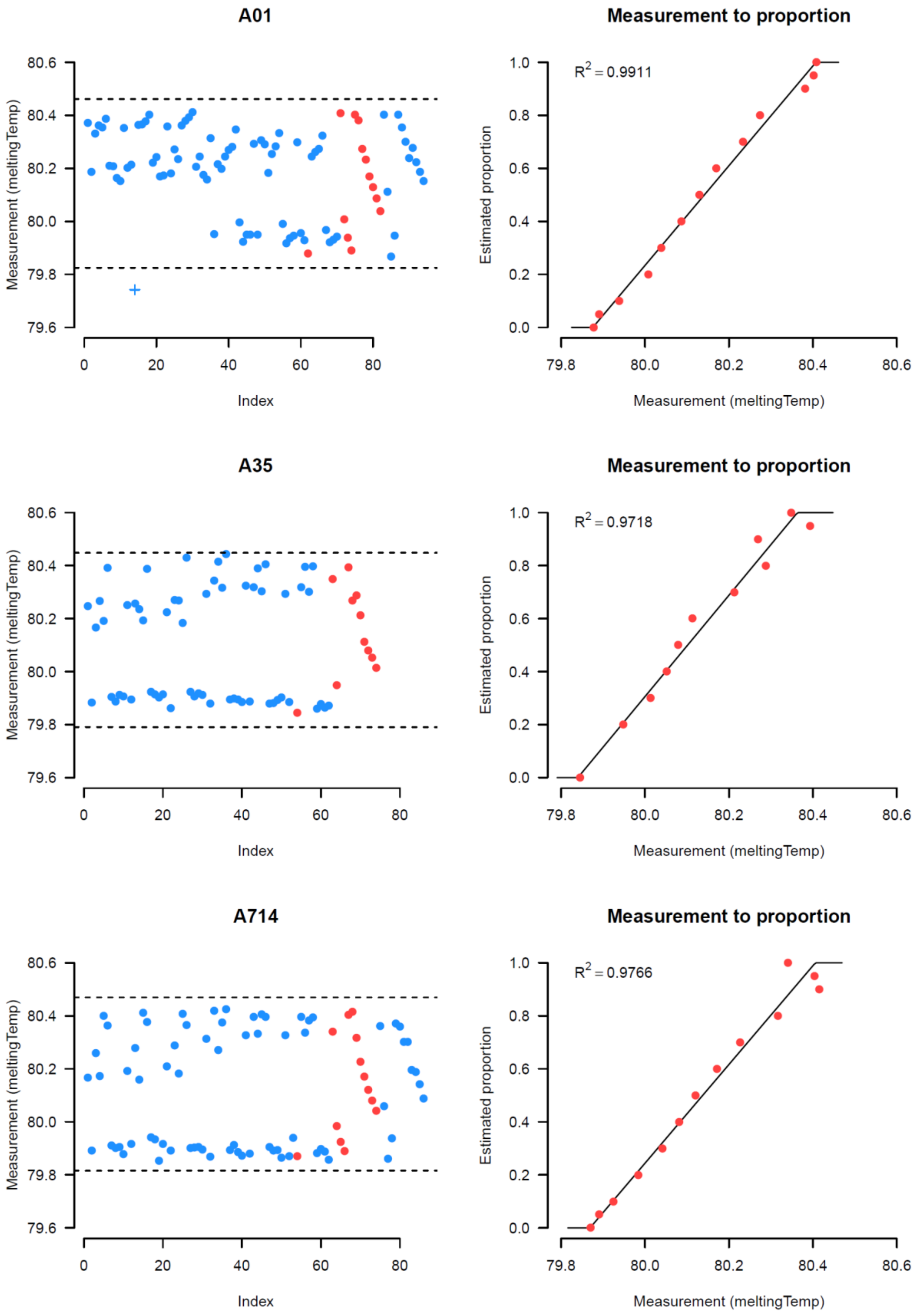

A01
Measurement to proportion
R2 = 0.9911
A35
Measurement to proportion
R2 = 0.9718
A714
Measurement to proportion
R2 = 0.9766
Measurement (meltingTemp)
Estimated proportion
Index
Measurement (meltingTemp)

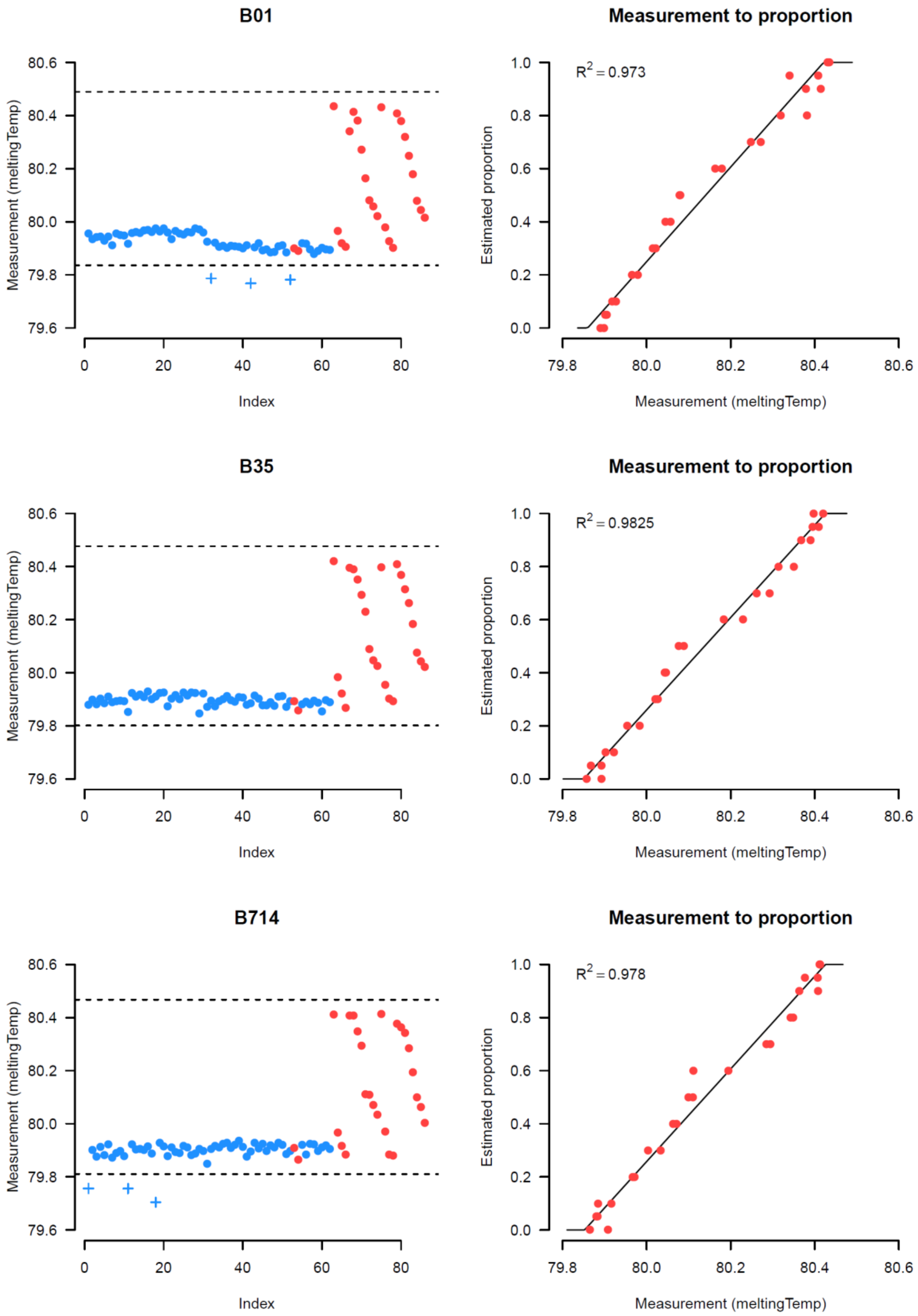
B01
Measurement to proportion
$R^2 = 0.973$
B35
Measurement to proportion
$R^2 = 0.9825$
B714
Measurement to proportion
$R^2 = 0.978$
Measurement (meltingTemp)
Index
Estimated proportion
Measurement (meltingTemp)
79.6
79.8
80.0
80.2
80.4
80.6
0
20
40
60
80
0.0
0.2
0.4
0.6
0.8
1.0

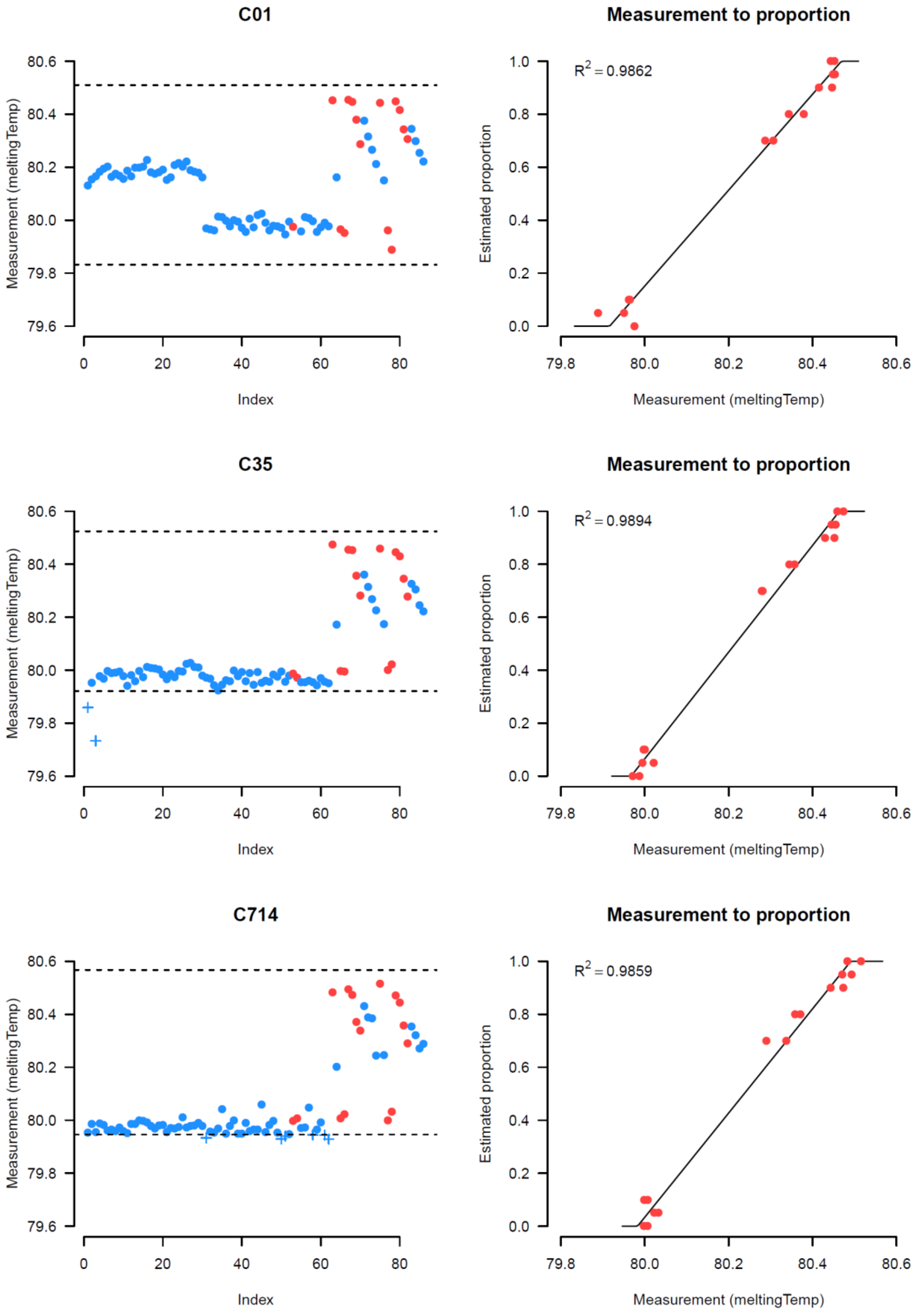
C01
Measurement to proportion
Measurement (meltingTemp)
Index
Estimated proportion
$R^2 = 0.9862$
C35
Measurement to proportion
Measurement (meltingTemp)
Index
Estimated proportion
$R^2 = 0.9894$
C714
Measurement to proportion
Measurement (meltingTemp)
Index
Estimated proportion
$R^2 = 0.9859$

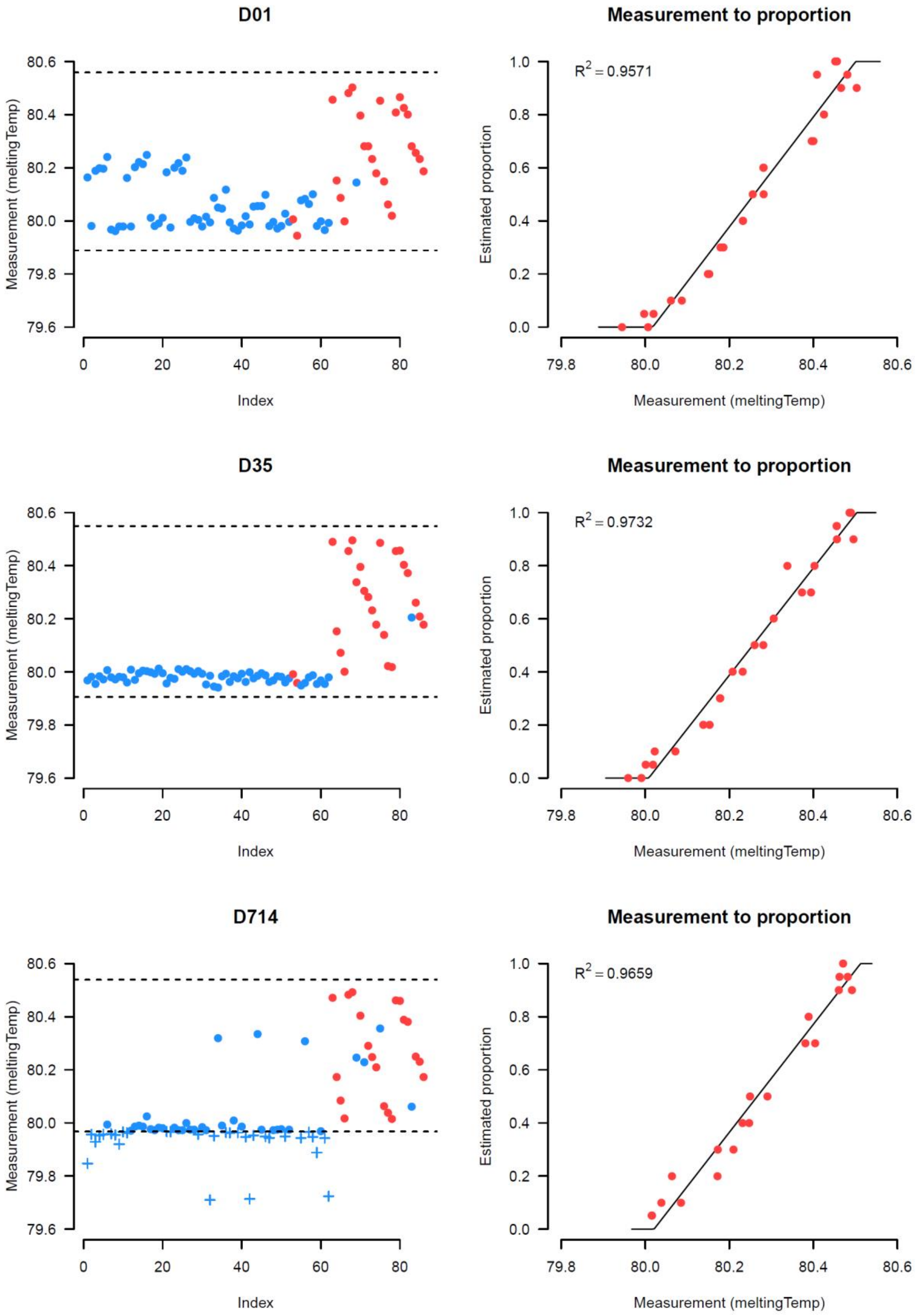
D01
Measurement (meltingTemp)
Index
Measurement to proportion
R2 = 0.9571
Estimated proportion
Measurement (meltingTemp)
D35
Measurement (meltingTemp)
Index
Measurement to proportion
R2 = 0.9732
Estimated proportion
Measurement (meltingTemp)
D714
Measurement (meltingTemp)
Index
Measurement to proportion
R2 = 0.9659
Estimated proportion
Measurement (meltingTemp)

Figure S4. (A) High-resolution melt normalization and **(B)** differential plots depicting four *Flavobacterium columnare* genotypes by HRMA (See the method in **Appendix S1.**)

(A)

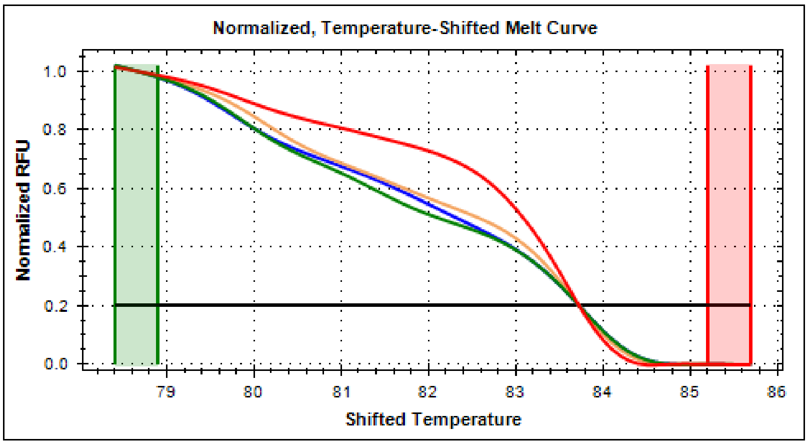

(B)

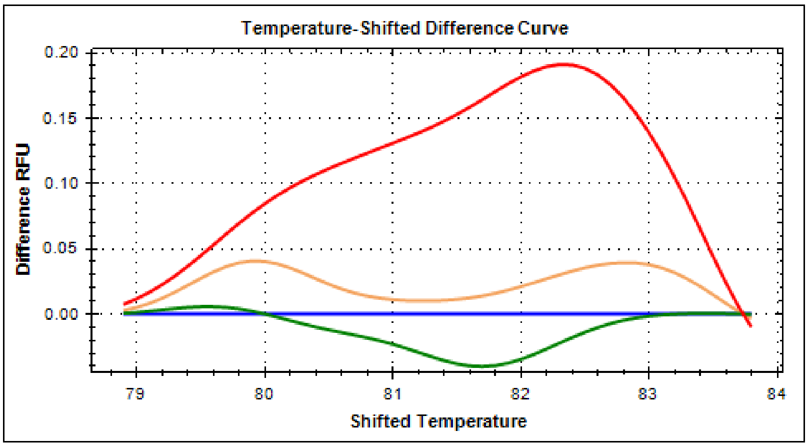

Appendix S1.

The duplex HRM assay developed for supplementry use in identification of *F. columnare* genotypes (C, E, G, A) that has been found in Finland so far. The PCR/HRM primers were designed to individually and simultaneously detect above genotypes. Primer sets have been designed that target different variable regions of *trpB* gene in *F. columnare*. The sequence of used primers was 5-CCGCATCATAGCCGAAACA-3 (CE-forward) and 5-GACGTTTGATGTCGATTTCGC-3 (CE-reverse reverse) for genotypes C and E amplification, and 5-CGCAGAAGTCCGTCCTG-3 (GA1-forward) and 5-AAAGGTATCTAGCGGATTGTT-3 (GA1-reverse) for genotypes G and A amplification. Primers amplified 113- and 97-bp products that corresponded to nucleotides 24 to 137 and 171 to 267, respectively, in *trpB* gene (GenBank accession numbers: LN624115 for genotype G, LN624121for genotype C, LN624122 for genotype A, LN624123 for genotype E)

Briefly, PCR amplification was performed in a total volume of 20 µL, containing 10ul of 1x Precision Melt Supermix (Bio-Rad Laboratories, USA), 1.2 µl of CE primer mix (final concentration 200 µm) primer, 0.8 µl of CE primer mix (final concentration 200nm), 3 µl of PCRgrade water and 5 µl 5 ng the DNA template . All samples were performed in triplicate. The PCR condition was started at 95°C for 1 minute for initial denaturation, followed by 40 cycles of 30 seconds at 95°C for denaturation, 30 seconds at 63°C for annealing and another 30 seconds at 72°C for extension. The PCR amplification was then followed by heteroduplex formation at 95°C for 30 seconds and subsequently 60°C for 1 minute. The high-resolution melting analysis was performed immediately afterwards by increasing the temperature from 65°C to 95°C by steps of 0.2°C maintained for 10 seconds each.

HRM analysis clearly separated 4 of aimed *F. columnare* genotypes with distinctive melting curves Supplementary_Figure 4.
